# Neuromyelitis Optica Spectrum Disorder With Anti-Aquaporin-4 Antibody: Outcome Prediction Models

**DOI:** 10.3389/fimmu.2022.873576

**Published:** 2022-03-31

**Authors:** Liang Wang, Lei Du, Qinying Li, Fang Li, Bei Wang, Yuanqi Zhao, Qiang Meng, Wenyu Li, Juyuan Pan, Junhui Xia, Shitao Wu, Jie Yang, Heng Li, Jianhua Ma, Jingzi ZhangBao, Wenjuan Huang, Xuechun Chang, Hongmei Tan, Jian Yu, Lei Zhou, Chuanzhen Lu, Min Wang, Qiang Dong, Jiahong Lu, Chongbo Zhao, Chao Quan

**Affiliations:** ^1^ Department of Neurology, Huashan Hospital, Shanghai Medical College, Fudan University, Shanghai, China; ^2^ National Center for Neurological Disorders (NCND), Shanghai, China; ^3^ Department of Neurology, The First Affiliated Hospital of Xinjiang Medical University, Urumqi, China; ^4^ Department of Rehabilitation Medicine, Jing’an District Centre Hospital of Shanghai, Fudan University, Shanghai, China; ^5^ Department of Rehabilitation Medicine, Huashan Hospital, Shanghai Medical College, Fudan University, Shanghai, China; ^6^ Department of Neurology, Jing’an District Centre Hospital of Shanghai, Fudan University, Shanghai, China; ^7^ Department of Neurology, The Second Affiliated Hospital of Guangzhou University of Chinese Medicine, Guangzhou, China; ^8^ Department of Neurology, The First People’s Hospital of Yunnan Province, Kunming, China; ^9^ Department of Neurology, Sir Run Run Shaw Hospital, School of Medicine, Zhejiang University, Hangzhou, China; ^10^ Department of Neurology, The First Affiliated Hospital of Wenzhou Medical University, Wenzhou, China; ^11^ Department of Neurology, The Fifth Affiliated Hospital of Zhengzhou University, Zhengzhou, China; ^12^ Department of Neurology, Wuhan No.1 Hospital, Wuhan, China; ^13^ Department of Neurology, Central Hospital Affiliated to Shandong First Medical University, Jinan, China; ^14^ Department of Ophthalmology and Vision Science, Eye and ENT Hospital, Shanghai Medical College, Fudan University, Shanghai, China

**Keywords:** neuromyelitis optica spectrum disorder, anti-aquaporin-4 antibody, maintenance therapy, outcome, prediction model

## Abstract

**Background:**

Recognizing the predictors of disease relapses in patients with anti-aquaporin-4 antibody (AQP4-ab)-positive neuromyelitis optica spectrum disorder (NMOSD) is essential for individualized treatment strategy. We aimed to identify the factors that predicted relapses among patients with AQP4-ab-positive NMOSD, develop outcome prediction models, and validate them in a multicenter validation cohort.

**Methods:**

Between January 2015 and December 2020, 820 patients with NMOSD were registered at Huashan Hospital. We retrospectively reviewed their medical records, and included 358 AQP4-ab-positive patients with 1135 treatment episodes. Univariate and multivariate analyses were used to explore the predictors of relapse, severe visual or motor disability during follow-up. A model predicting the 1- and 2-year relapse-free probability was developed and validated in an external validation cohort of 92 patients with 213 treatment episodes.

**Results:**

Lower serum AQP4-ab titer (< 1:100), higher Expanded Disability Status Scale (EDSS) score at onset (≥ 2.5), and use of intravenous methylprednisolone (IVMP) at the first attack predicted an overall lower annualized relapse rate. Older age (> 48 years), optic neuritis at onset, and higher onset EDSS score (≥ 2.5) significantly increased the risk for blindness, while IVMP at the first attack and maintenance therapy reduced the risk for blindness. Myelitis at onset increased the possibility of motor disability (EDSS ≥ 6.0), severe motor disability or death (EDSS ≥ 8.0), while maintenance therapy reduced these possibilities. Anderson and Gill model identified that the risk factors predicting recurrent relapses under certain treatment status were female gender, high AQP4-ab titer (≥ 1:100), previous attack under same therapy, lower EDSS score at treatment initiation (< 2.5), and no maintenance therapy or oral prednisone lasting less than 6 months. A nomogram using the above factors showed good discrimination and calibration abilities. The concordance indexes in the primary and validation cohort were 0.66 and 0.65, respectively.

**Conclusion:**

This study reports the demographic, clinical and therapeutic predictors of relapse, and severe visual or motor disability in NMOSD. Early identification of patients at risk of unfavorable outcomes is of paramount importance to inform treatment decisions.

## Introduction

Neuromyelitis optica spectrum disorders (NMOSD) are autoantibody-induced inflammatory diseases of the central nervous system, characterized by recurrent optic neuritis (ON) and transverse myelitis (TM), leading to blindness and paralysis (1). Specific serum anti-aquaporin-4 antibodies (AQP4-ab) are pathogenic and identified in most patients with NMOSD (2). A proportion of AQP4-ab-negative NMOSD patients have serum anti-myelin oligodendrocyte glycoprotein antibodies (MOG-ab) and exhibit different characteristics than AQP4-ab-positive patients (3).

Disability in NMOSD patients depends on the relapses; therefore, the primary goal of NMOSD treatment is to prevent or delay attacks. Drugs conventionally used as maintenance therapy in NMOSD include immunosuppressants and monoclonal antibodies, such as azathioprine (AZA), mycophenolate mofetil (MMF), and rituximab (RTX) (4). Recent randomized controlled trials of satralizumab, eculizumab, and inebilizumab have reported promising results for NMOSD treatment (5–8). However, consensus regarding the position of these new preventive drugs in the management algorithm has not been reached. There is insufficient evidence to compare the effectiveness of different maintenance therapies for NMOSD (9, 10). Little is known about the demographic, clinical, and therapeutic predictors of relapse, severe visual or motor disability in NMOSD (11–16). Early identification of patients at risk of unfavorable outcomes is critical for individualized treatment decisions.

The aim of the present study was to investigate the treatment outcomes of NMOSD in China, compare the effectiveness of different maintenance therapies by analyzing the relapses, evaluate the risk factors that predict relapse, and severe visual or motor disability, and design a nomogram that can be used to estimate the probability of 1- and 2-year relapse-free status.

## Methods

### Study Design and Participants

This retrospective cohort study involved a review of the medical records of 820 consecutive NMOSD patients who presented to the Shanghai Huashan Hospital, China, between January 2015 and December 2020. Patients were included to form a primary cohort in this study if they fulfilled the criteria established by the International Panel for NMOSD diagnosis in 2015 (17), were serum AQP4-ab-positive by cell-based assays, were followed up for at least 2 years, and had treatment episodes for at least 3 months, with definite start and stop dates.

We collected the demographic and clinical data of eligible patients, including onset age, gender, serum AQP4-ab titer, onset attack type (ON, TM, brainstem/cerebral, or mixed attack), subsequent attack (type, start date, treatment, and outcome), Expanded Disability Status Scale (EDSS) score, visual acuity, maintenance therapy (dosage, start and stop dates), and concomitant auto-antibodies. The first available serum AQP4-ab titer detected in a remission status was used in this analysis.

An attack/relapse was defined as worsening of existing symptoms, or occurrence of new symptoms, lasting for at least 24 hours; multiple symptoms within 30 days were regarded as a single attack (18). We defined “first attack” as the inaugural attack that marked the disease onset, “first relapse” as the second attack experienced after the disease onset, and recurrent relapses as all attacks after the first attack.

We identified the predictors of annualized relapse rates (ARRs; calculated from disease onset to end of follow-up, excluding the first attack) for ON, TM, brainstem/cerebral attacks, and overall attacks. We also evaluated the risk factors for first relapse, blindness (visual acuity ≤ 0.1 for > 6 months), motor disability (EDSS score ≥ 6.0), and severe disability or death (EDSS score ≥ 8.0).

To explore the predictors for recurrent relapses despite maintenance treatment, the duration was calculated from the initiation of certain maintenance therapy to the subsequent endpoint. Patients who received no treatment were also recognized as a treatment category and included in the primary cohort. The predictors identified from the primary cohort were validated on an external validation cohort, consisting of 92 AQP4-ab-positive patients from Jing’an District Central Hospital (n = 26), the First Affiliated Hospital of Xinjiang Medical University (n = 18), the Second Affiliated Hospital of Guangzhou University of Chinese Medicine (n = 17), the First People’s Hospital of Yunnan Province (n = 9), Sir Run Run Shaw Hospital (n = 9), the First Affiliated Hospital of Wenzhou Medical University (n = 5), the Fifth Affiliated Hospital of Zhengzhou University (n = 3), Wuhan First Hospital (n = 3), and Jinan Central Hospital (n = 2).

All patients from the Huashan Hospital and nine other study centers underwent serum AQP4-ab and MOG-ab detection with fixed cell-based indirect immune-fluorescence tests. HEK293 cells transfected with either the M1 isoform of AQP4 or full-length human MOG were used. AQP4-ab titer ≥ 1:100 was defined as a high AQP4-ab level. All AQP4-ab-positive patients were not found to harbor MOG-ab.

### Treatment Episodes

NMOSD treatments during follow-up were divided into stable (no relapse) and failed (disease relapse) treatment episodes. The effectiveness durations of medications from the time of last administration were as follows: 180 days for RTX; 90 days for mitoxantrone (MIT); 30 days for AZA, MMF, tacrolimus (TAC), cyclophosphamide (CTX), cyclosporine A (CsA), intravenous methylprednisolone (IVMP), intravenous immunoglobulin (IVIG), and plasma exchange (PE); and 7 days for methotrexate (MTX) and oral steroids (11). Treatment episodes were excluded from analyses if they consisted of less than 15 episodes of a certain medication, lasted for < 3 months, without documented start or stop dates, or consisted of patients who had double/overlapping treatments. The duration of certain treatment episodes was prolonged, if there was no relapse. In case of gaps between treatment episodes that lasted shorter than the effectiveness duration, the episodes were considered a single episode.

### Statistical Analyses

We performed statistical analyses and constructed figures using the R software (version 4.0.3; R Foundation for Statistical Computing, Vienna, Austria; http://www.r-project.org/), using rms, survival, and ggDCA packages (19–21). Continuous variables are presented as medians (interquartile range, IQR) or means (± 1 standard deviation, SD).

Two datasets were used for the analyses. In Dataset A, generalized linear regression was applied to evaluate the associations between multiple variables and ARRs for ON, TM, brainstem/cerebral and all attack types. Univariate cox regression was applied to analyze the associations of multiple variables with first relapse, blindness, disability, and severe disability. Maintenance therapy was selected as a time-dependent variable. In Dataset B, we selected patient identification as a cluster variable in the Anderson-Gill (AG) proportional-hazards model, and analyzed recurrent events in terms of “time to subsequent relapse” (22). Variables associated with significant rate ratios (RRs) or hazard ratios (HRs) (i.e., p < 0.1) in the univariate analysis were further analyzed with a multivariate model.

Some events were analyzed together to improve statistical power and stability. We analyzed unilateral and bilateral ON, brainstem attacks, and cerebral attacks together because the number of cerebral attacks was insufficient. Similarly, IVMP, IVIG, and PE were combined as acute attack therapy; unilateral and bilateral blindness as well as EDSS score ≥ 8.0 and death were combined as the composite endpoint. If a patient had a mixed attack of ON and TM, the attack would contribute to the ARRs for both ON and TM.

A model was developed to predict disease relapses using the AG model; this model was depicted in a nomogram to estimate the 1- and 2-year relapse-free probability in the primary cohort and validated in the external cohort. Concordance index (C-index), calibration plot, and decision curve analysis (DCA) were used to evaluate the discrimination and calibration ability of the model, as well as its clinical usefulness. Bootstrap with 1000 resampling was performed for the internal and external validation in the primary and validation cohorts, respectively, using the C-index and calibration curve (23, 24). Statistical significance was set at p-value < 0.05.

### Data Availability

Anonymized data not presented in the study will be made available upon request from any qualified investigator.

## Results

### Cohort Study

**Figure 1:**
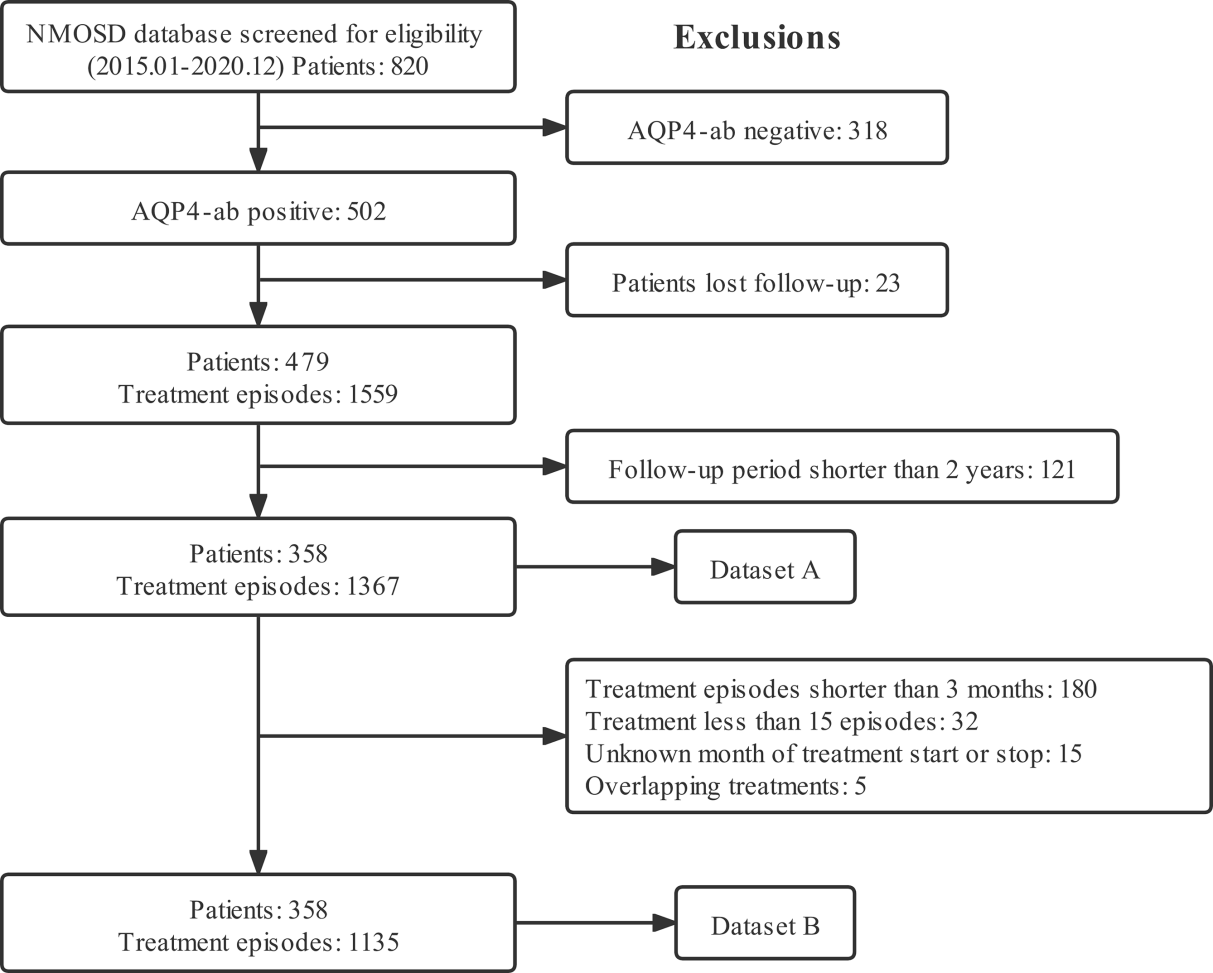
Flow chart for data inclusion and the exclusion criteria. Dataset A was analyzed for descriptive statistics, ARRs, and clinical events. Dataset B was analyzed for recurrent events in terms of time to subsequent relapse. ARR, annualized relapse rate; NMOSD, neuromyelitis optica spectrum disorder; AQP4-ab, anti-aquaporin-4 antibody.

**Figure 1** depicts the flow chart for data inclusion and exclusion. We included 358 patients with 1135 treatment episodes from Huashan Hospital to form the primary cohort, and 92 patients with 213 treatment episodes from other centers to form the external validation cohort. Baseline demographic and clinical characteristics of the two cohorts were comparable (**Table 1**). The most common onset manifestations were ON and TM; AZA, MMF, and RTX were the most frequently prescribed immunosuppressive drugs.

**Table 1:** Baseline demographic and clinical characteristics of AQP4-ab positive NMOSD patients.

Patient characteristics	Primary cohort	Validation cohort
Patient number	358	92
Female gender	329 (91.9)	84 (91.3)
Age at disease onset (years)	36.2 ± 14.7	45.3 ± 14.7
Disease duration (months)	61.9 (41.7-100.5)	54.9 (39.8-76.1)
AQP4-ab titer	1:100 (1:32-1:100)	1:100 (1:32-1:320)
ARR at final visit	0.5 (0.3-0.7)	0.4 (0.3-0.7)
ARR-1 at final visit	0.3 (0.2-0.5)	0.2 (0-0.4)
Baseline EDSS score	1 (0–2)	2 (1-3)
Initial manifestation		
ON	162 (45.3)	36 (39.1)
TM	155 (43.3)	67 (72.8)
Brainstem syndrome	105 (29.3)	10 (10.9)
Cerebral syndrome	4 (1.1)	0 (0)
Pregnancy within study period	43 (12.0)	2 (2.2)
Maintenance therapy	Treatment episodes, n (patients exposed, n)	
Prednisone (≥ 6 months)	23 (19)	10 (8)
AZA	227 (176)	50 (29)
MMF	185 (142)	28 (25)
TAC	48 (40)	1 (1)
RTX	96 (70)	19 (16)
CTX	24 (23)	7 (7)
CsA	3 (3)	2 (2)
MTX	2 (2)	0 (0)
MIT	4 (4)	0 (0)
IFN	7 (3)	0 (0)
ASCT	3 (2)	0 (0)
Acute attack therapy	Treatment episodes, n (patients exposed, n)	
IVMP	937 (355)	192 (87)
IVIG	176 (130)	18 (18)
PE	23 (21)	2 (2)

Numerical data are n (%), mean ± SD, or median (IQR). AQP4-ab, anti-aquaporin-4 antibody; ARR, annualized relapse rate; EDSS, Expanded Disability Status Scale; ON, optic neuritis; TM, transverse myelitis; AZA, azathioprine; MMF, mycophenolate mofetil; TAC, tacrolimus; RTX, rituximab; CTX, cyclophosphamide; CsA, cyclosporin A; MTX, methotrexate; MIT, mitoxantrone; IFN, interferon; ASCT, autologous stem cell transplantation; IVMP, intravenous methylprednisolone; IVIG, intravenous immunoglobulin; PE, plasma exchange.

### Predictors of ARRs

The ARRs for each attack type (ON, TM, or brainstem/cerebral attacks) and overall ARRs covering all attack types were calculated. To avoid over-estimating the ARR, the inaugural attack that defined the disease onset was excluded from the calculation. **Supplementary Table 1** and **Table 2** summarize the effects of predictors on ARRs and certain events in the univariate and multivariate analyses. Patients with high AQP4-ab levels (≥ 1:100) had higher ARRs for TM (p = 0.038) and all attacks (p = 0.016) compared to those with low AQP4-ab levels (< 1:100). Patients with ON at onset had higher ARRs for ON (p < 0.001), while those with TM at onset had lower ARRs for brainstem/cerebral attacks (p < 0.001). Patients with higher EDSS score at onset (≥ 2.5) had lower ARRs for TM (p = 0.003), and all attacks (p = 0.025). Compared to patients not treated with corticosteroids, those treated with IVMP at disease onset had ARRs reduced by 9% for ON (p < 0.001), and 9% for all attacks (p = 0.005). An inverse correlation was observed between maintenance therapy and all attacks (p = 0.18), albeit without statistical significance.

**Table 2:** Estimation of the effects of predictors on ARRs with multivariate analysis.

Predictors	ON		TM		Brainstem/Cerebral		All	
	Rate ratio	p-value	Rate ratio	p-value	Rate ratio	p-value	Rate ratio	p-value
Female gender (Reference = male)	—	—	—	—	—	—	1.08 (0.97-1.19)	0.17
AQP4-ab titer (Reference = < 1:100)	1.04 (1.00-1.08)	0.073	1.05 (1.00-1.09)	0.038*	—	—	1.07 (1.01-1.13)	0.016*
Onset age, years (Reference = > 48)								
≤ 35	—	—	—	—	1.02 (0.98-1.06)	0.29	1.03 (0.96-1.11)	0.36
35-48	—	—	—	—	0.99 (0.95-1.03)	0.47	1.05 (0.96-1.13)	0.29
Onset attack (Reference = brainstem/cerebral)								
ON	1.16 (1.09-1.23)	< 0.001***	—	—	0.97 (0.93-1.01)	0.11	—	—
TM	1.02 (0.96-1.09)	0.54	—	—	0.92 (0.88-0.96)	< 0.001***	—	—
Mixed	1.06 (0.99-1.14)	0.094	—	—	0.98 (0.93-1.03)	0.43	—	—
Concomitant auto-antibodies (Reference = < 1)	0.96 (0.93-1.00)	0.072	—	—	—	—	—	—
Concomitant auto-antibodies (Reference = < 2)	—	—	—	—	—	—	—	—
Onset EDSS score (Reference = < 2.5)	—	—	0.93 (0.88-0.97)	0.003**	0.96 (0.93-1.00)	0.053	0.92 (0.86-0.99)	0.025*
IVMP at the first attack (Reference = no)	0.91 (0.86-0.96)	< 0.001***	—	—	—	—	0.91 (0.85-0.97)	0.005**
Maintenance therapy (Reference = no or prednisone < 6 months)	—	—	—	—	—	—	—	—

AQP4-ab, anti-aquaporin-4 antibody; ON, optic neuritis; TM, transverse myelitis; EDSS, Expanded Disability Status Scale; IVMP, intravenous methylprednisolone. *p < 0.05, **p < 0.01, ***p < 0.001.

### Predictors of First Relapse, Blindness, Motor Disability, and Severe Disability During Follow-Up

We explored the factors that predicted first relapse, blindness, disability, and severe disability during follow-up. Patients with younger onset age (≤ 35), compared to those with older onset age, had lower risk for blindness (p = 0.014). Patients with ON at onset had significantly higher risk for blindness (p = 0.002), while patients with TM at onset had significantly higher risk for disability and severe disability (p = 0.004 and 0.043, respectively). Patients with higher EDSS score at disease onset (≥ 2.5) had higher risk for blindness (p < 0.001). IVMP treatment at the first attack and maintenance therapy significantly reduced the risk for first relapse (p = 0.026 and p < 0.001, respectively), blindness (p = 0.034 and p < 0.001, respectively), while only maintenance therapy significantly lowered the risk for disability and severe disability during follow-up (p < 0.001 and p = 0.005, respectively) (**Supplementary Table 2** and **Table 3**).

**Table 3:** Estimation of the effects of predictors on certain events with multivariate analysis.

Predictors	First relapse		Blindness		EDSS score ≥ 6.0		EDSS score ≥ 8.0/Death	
	Hazard ratio	p-value	Hazard ratio	p-value	Hazard ratio	p-value	Hazard ratio	p-value
Female gender (Reference = male)	—	—	—	—	—	—	—	—
AQP4-ab titer (Reference = < 1:100)	—	—	—	—	—	—	—	—
Onset age, years (Reference = > 48)								
≤ 35	—	—	0.51 (0.30-0.87)	0.014*	0.75 (0.50-1.13)	0.17	0.65 (0.35-1.20)	0.17
35-48	—	—	0.67 (0.36-1.24)	0.20	0.76 (0.48-1.22)	0.26	0.76 (0.39-1.49)	0.42
Onset attack (Reference = brainstem/cerebral)								
ON	0.97 (0.68-1.39)	0.88	3.69 (1.59-8.58)	0.002**	0.65 (0.38-1.09)	0.10	0.60 (0.24-1.50)	0.27
TM	1.04 (0.71-1.52)	0.83	0.77 (0.27-2.20)	0.62	2.08 (1.27-3.41)	0.004**	2.30 (1.03-5.16)	0.043*
Mixed	1.00 (0.66-1.52)	0.99	0.90 (0.32-2.52)	0.84	1.46 (0.84-2.53)	0.18	2.09 (0.87-5.03)	0.10
Concomitant auto-antibodies (Reference = < 1)	—	—	—	—	1.49 (1.06-2.09)	0.022*	—	—
Concomitant auto-antibodies (Reference = < 2)	0.80 (0.61-1.04)	0.096	—	—	—	—	—	—
Onset EDSS score (Reference = < 2.5)	0.77 (0.56-1.06)	0.11	7.13 (4.10-12.39)	< 0.001***	—	—	1.53 (0.83-2.82)	0.18
IVMP at the first attack (Reference = no)	0.72 (0.53-0.96)	0.026*	0.58 (0.35-0.96)	0.034*	—	—	—	—
Maintenance therapy (Reference = no or prednisone < 6 months)	0.25 (0.15-0.40)	< 0.001***	0.17 (0.08-0.37)	< 0.001***	0.12 (0.06-0.23)	< 0.001***	0.35 (0.17-0.73)	0.005**

AQP4-ab, anti-aquaporin-4 antibody; ON, optic neuritis; TM, transverse myelitis; EDSS, Expanded Disability Status Scale; IVMP, intravenous methylprednisolone. *p < 0.05, **p < 0.01, ***p < 0.001.

### Predictors of Recurrent Relapses and Development of Individualized Prediction Model

Recurrent relapses were defined as all attacks after the first attack as mentioned above. We explored the predictors of recurrent relapses under certain treatment episode. The analysis included 358 patients with 1135 treatment episodes. **Supplementary Figure 1** is a forest plot for predictors of recurrent relapses during treatment episodes, identified using a univariate AG model. We incorporated treatment episodes with no treatment or prednisone < 6 months as the same category for the HR was 1.01 (95% confidence interval [CI]: 0.81–1.27) (p = 0.92).

Multivariate AG analysis identified that the risk factors predicting recurrent relapses were female gender, high AQP4-ab titer (≥ 1:100), previous attack under same therapy, lower EDSS score at treatment initiation (< 2.5), and no maintenance therapy or oral prednisone lasting less than 6 months (**Table 4**). We also observed that RTX showed the best effect in preventing relapses (HR = 0.20, p < 0.001), while CTX was the weakest among various drugs (HR = 0.86, p = 0.71), with reference to no maintenance drug or use of oral prednisone less than 6 months.

**Table 4:** Independent predictors of recurrent relapse with multivariate Anderson and Gill model.

Independent predictors	Model	
	Hazard ratio (95% CI)	p-value
Female gender (Reference = male)	1.37 (1.01-1.86)	0.042*
AQP4-ab titer (Reference = < 1:100)	1.28 (1.06-1.55)	0.010*
Previous attack under same therapy (Reference = no)	1.32 (1.08-1.61)	0.007**
EDSS score at treatment initiation (Reference = < 2.5)	0.80 (0.66-0.97)	0.023*
Maintenance therapy (Reference=no or prednisone < 6 months)		
Prednisone (≥ 6 months)	0.29 (0.14-0.59)	< 0.001***
AZA	0.42 (0.32-0.56)	< 0.001***
MMF	0.35 (0.24-0.50)	< 0.001***
TAC	0.37 (0.21-0.64)	< 0.001***
RTX	0.20 (0.11-0.36)	< 0.001***
CTX	0.86 (0.38-1.92)	0.71
C-index		
Primary cohort	0.66 (0.64-0.69)	
Validation cohort	0.65 (0.60-0.70)	

AQP4-ab, aquaporin-4 antibody; EDSS, Expanded Disability Status Scale; AZA, azathioprine; MMF, mycophenolate mofetil; TAC, tacrolimus; RTX, rituximab; CTX, cyclophosphamide. *p < 0.05, **p < 0.01, ***p < 0.001.

**Figure 2:**
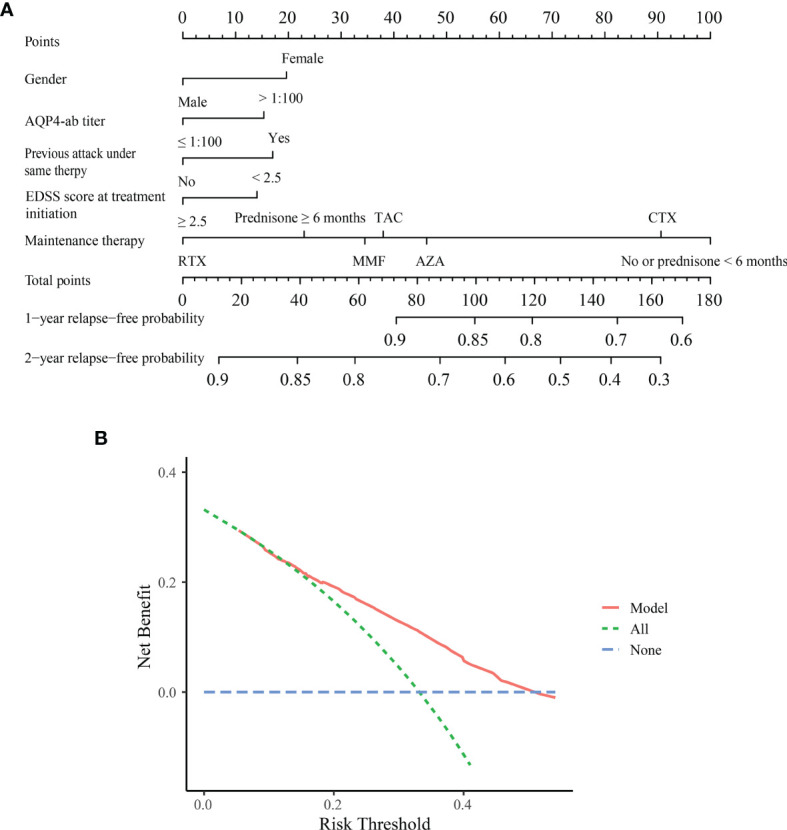
Nomogram and decision curve analysis in the primary cohort. **(A)** Nomogram for predicting 1- and 2-year relapse-free probability in the primary cohort. Based on each variable axis of the nomogram, the probability for each patient was determined (upward-pointing line). At the total-points axis, the sum of points corresponds to 1- and 2-year relapse-free probability (downward-pointing line). **(B)** Decision curve analysis for the Model. The decision curve revealed that if the threshold probability exceeds 0.15, this nomogram was superior for predicting subsequent relapses compared to either treat-all or treat-none scheme. ON, optic neuritis; TM, transverse myelitis; EDSS, Expanded Disability Status Scale; AZA, azathioprine; MMF, mycophenolate mofetil; TAC, tacrolimus; RTX, rituximab; CTX, cyclophosphamide.

**Figure 3:**
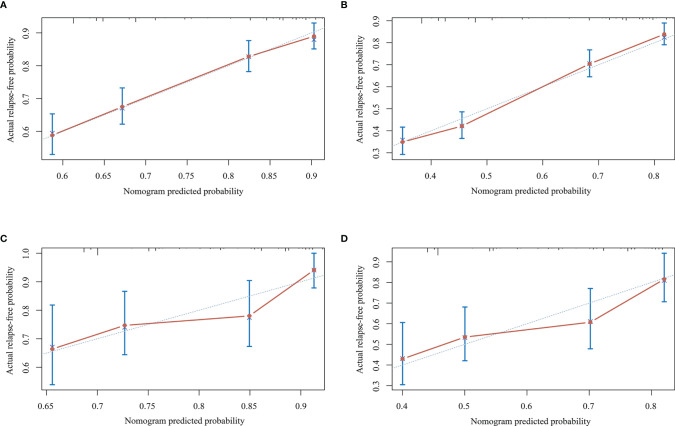
Calibration curves for the nomogram in the primary and validation cohorts. **(A)** Calibration curve for predicting the 1-year relapse-free probability in the primary cohort. **(B)** Calibration curve for predicting 2-year relapse-free probability in the primary cohort. **(C)** Calibration curve for predicting 1-year relapse-free probability in the validation cohort. **(D)** Calibration curve for predicting 2-year relapse-free probability in the validation cohort.

**Figure 2A** depicts the nomogram for this Model, including the above factors used to estimate the 1- and 2-year relapse-free probability. The calibration curve demonstrated good agreement between the predicted and actual 1- and 2-year relapse-free probability in the primary cohort (**Figures 3A, B**). The C-index of the nomogram was 0.66 (95% CI: 0.64–0.69) and 0.68 *via* bootstrapping validation. **Figure 2B** depicts the DCA for this Model. The decision curve demonstrated that for threshold probability exceeding 0.15, the nomogram was associated with improved prediction of subsequent relapses and was superior to the treat-all or treat-none strategy.

### External Validation of the Prediction Model for Recurrent Relapses

The prediction model for recurrent relapses during certain treatment episode was validated on an external cohort. In the validation cohort, good calibration was observed between the predicted and actual 1- and 2-year relapse-free probability (**Figures 3C, D**). The C-index of the nomogram was 0.65 (95% CI: 0.60–0.70) and 0.70 with bootstrap resampling.

## Discussion

Previous studies have reported the predictors of relapse, blindness, and disability in NMOSD patients using univariate and multivariate regression analysis (11, 14, 25–28). The current study included the largest multicenter cohort of AQP4-ab-positive patients to date and systematically explored the predictors of relapse, and severe visual or motor disability in NMOSD. We demonstrated that independent factors predicting recurrent relapses in NMOSD included female gender, high AQP4-ab titer (≥ 1:100), previous attack under same therapy, lower EDSS score at treatment initiation (< 2.5), and no maintenance therapy or oral prednisone lasting less than 6 months. We also developed a feasible prediction model that could facilitate the selection of appropriate immunotherapy to reduce the probability of subsequent relapse.

We investigated the use of immunotherapies for AQP4-ab-positive patients in China. AZA, MMF, and RTX were the most commonly used first-line immunosuppressive therapies that decrease relapses, consistent with the previous study (9). We also observed that no or inappropriate immunotherapy was used for some NMOSD patients (e.g., use of interferon in NMOSD patients misdiagnosed with multiple sclerosis), indicating antibody detection should be considered as an indispensable examination to improve the diagnostic accuracy of central nervous system idiopathic inflammatory demyelinating diseases.

Previous studies have evaluated factors associated with ARRs and certain events such as first relapse, blindness, and disability in NMOSD (14, 25, 28). These studies reported that onset age, onset attack type, and maintenance therapy were associated with ARRs and disability events, similar to our results. Older patients were found more likely to develop blindness and disability compared to younger patients, which suggested that late-onset NMOSD had a worse prognosis. Seok et al. evaluated the clinical characteristics of late- and early-onset NMOSD and observed that onset age had a significant positive correlation with EDSS score (29). In our study, older onset age was associated with a higher probability of blindness. Additionally, our results emphasized the importance of acute attack therapy, which significantly decreased the risk for first relapse and blindness. As researchers pointed out that IVMP should be administered within 4 days of attack onset for full visual recovery (30).

We evaluated the predictors for recurrent relapses in the study cohort. Time to first relapse under certain treatment was the most commonly used study outcome measure; however, few studies focused on the predictors of recurrent NMOSD events using time to subsequent relapse (26, 31). Consistent with previous reports (11, 26), we found that maintenance therapy predicted subsequent relapse, using a multivariate AG model. RTX was the most potent immunotherapy, consistent with the previous studies that compared the first-line therapies (AZA, MMF, and RTX) (9). The current study included additional immunotherapies (such as prednisone ≥ 6 months, TAC, CTX, and CsA) to provide a more comprehensive comparison. Stellmann et al. reported that previous attacks on the same treatment regimen increase the probability of future relapses, but without any statistical significance (11). In this study, we found this variable to be a strong predictor of future relapses (HR 1.32; p=0.007); therefore, we recommend that patients with attacks on certain treatment should switch to a more potent immunotherapy drug, such as RTX, without delay.

The prediction model was simple and feasible to use, especially with a nomogram that predicted the probability of subsequent relapses. Nomograms are widely used to determine the prognosis in the fields of medicine and oncology (32). The nomogram developed from the primary cohort and the calibration curve exhibited good agreement, while the C-index demonstrated good discrimination ability in the internal and external cohort (0.66 and 0.65). The significance of this nomogram was that it provided an effective reference regarding the prognosis when counseling NMOSD patients about their risk for subsequent relapses. To justify the use of the nomogram, we evaluated the effects of the nomogram on patient outcomes. The novel DCA method uses threshold probability to determine the clinical outcomes and the corresponding net benefit. The nomogram was found to be clinically useful in the DCA.

The primary limitation of this study was its retrospective nature, with the associated possibility of recall bias. The dose of maintenance therapies was not standardized in the primary and validation cohorts. Additionally, the AQP4-ab titers were not determined at fixed time points after NMOSD onset. Future prospective studies, with larger sample sizes and scheduled timings of antibody detection, are needed to validate our findings.

To conclude, this study evaluated the demographic, clinical and therapeutic predictors of relapse, and severe visual or motor disability in NMOSD. Early identification of the patients with unfavorable outcome features is of paramount importance to inform treatment decisions.

## Data Availability Statement

The raw data supporting the conclusions of this article will be made available by the authors, without undue reservation.

## Ethics Statement

The studies involving human participants were reviewed and approved by HIRB-2020007. The patients/participants provided their written informed consent to participate in this study.

## Author Contributions

LW, LD and QL designed and conceptualized the study, interpreted and analyzed the data, drafted and revised the manuscript for intellectual content. FL, BW, YZ, QM, WL, JP, JX, SW, JYa, HL, JM provided the data of the validation cohort. JZB, WH, XC, HT, JYu and LZ provided the data of the primary cohort and revised the manuscript for intellectual content. CL, MW, QD, JL, and CZ revised the manuscript for intellectual content. CQ designed and conceptualized the study, interpreted and analyzed the data, and revised the manuscript for intellectual content. Statistical analyses of this manuscript were conducted by LW and CQ. The corresponding author had full access to all the data in the study and had final responsibility for the decision to submit for publication.

## Funding

This research was supported by the National Natural Science Foundation of China (Grant No. 82171341, 81771296), the Shanghai Municipal Science and Technology Major Project (No. 2018SHZDZX01) and ZHANGJIANG LAB, and the National Key Research and Development Program of China (2016YFC0901504).

## Conflict of Interest

The authors declare that the research was conducted in the absence of any commercial or financial relationships that could be construed as a potential conflict of interest.

## Publisher’s Note

All claims expressed in this article are solely those of the authors and do not necessarily represent those of their affiliated organizations, or those of the publisher, the editors and the reviewers. Any product that may be evaluated in this article, or claim that may be made by its manufacturer, is not guaranteed or endorsed by the publisher.

## Supplementary Material

Supplementary Figure 1 Forest plot of predictors of recurrent relapse with univariate Anderson, and Gill model. AQP4-ab, anti-aquaporin-4 antibody; ON, optic neuritis; TM, transverse myelitis; EDSS, Expanded Disability Status Scale; ARR, annualized relapse rate; IVMP, intravenous methylprednisolone; IVIG, intravenous immunoglobulin; PE, plasma exchange; AZA, azathioprine; MMF, mycophenolate mofetil; TAC, tacrolimus; RTX, rituximab; CTX, cyclophosphamide. *p < 0.05, **p < 0.01, ***p < 0.001.
