# Supplementary figures and images for: Neuromyelitis Optica Spectrum Disorder With Anti-Aquaporin-4 Antibody: Outcome Prediction Models
Source: Front Immunol. 2022 Mar 31;13:873576. doi: 10.3389/fimmu.2022.873576 (PMC9012141; doi:10.3389/fimmu.2022.873576)

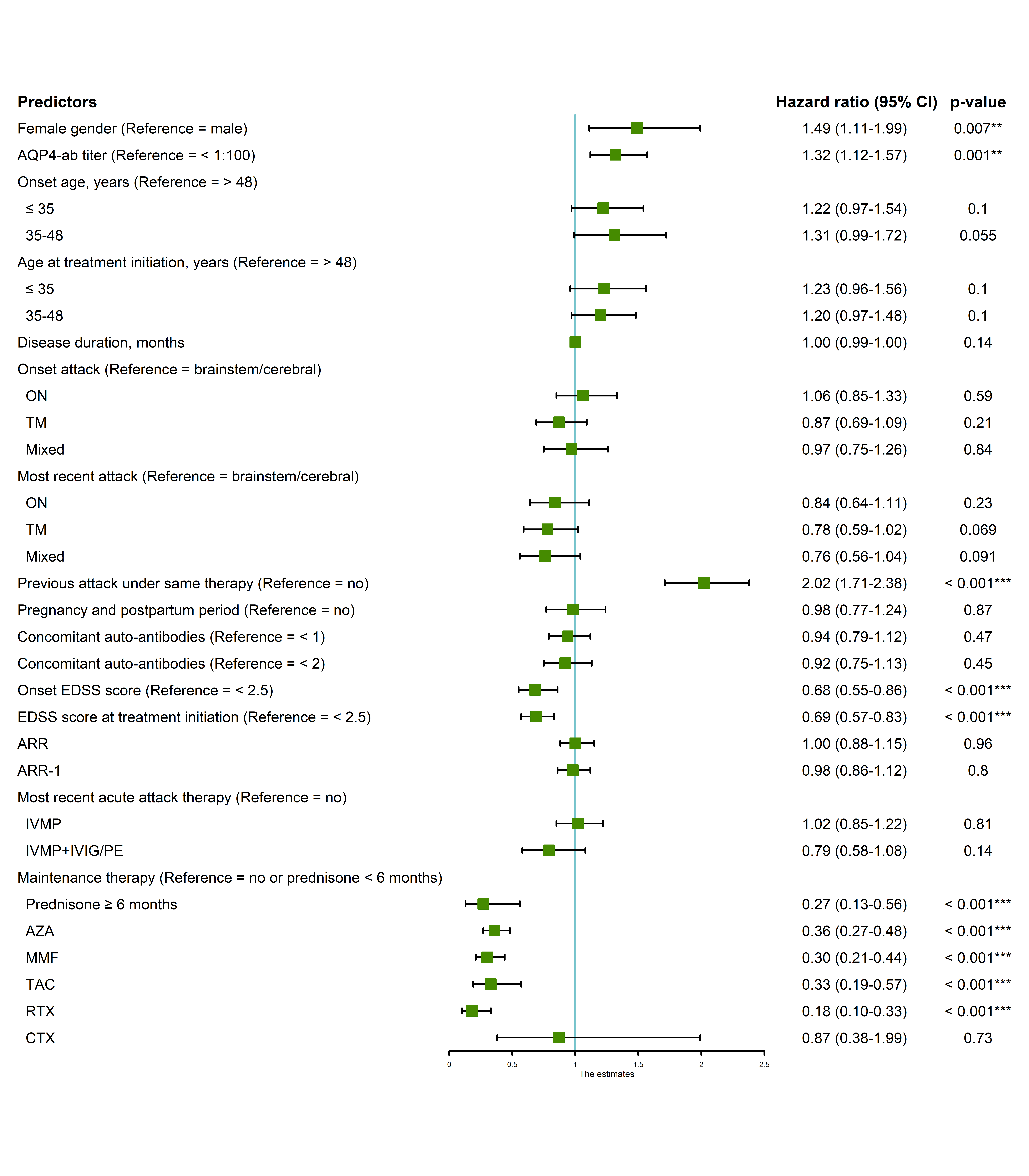

Supplement: Supplementary Figure 1 — Forest plot of predictors of recurrent relapse with univariate Anderson, and Gill model. AQP4-ab, anti-aquaporin-4 antibody; ON, optic neuritis; TM, transverse myelitis; EDSS, Expanded Disability Status Scale; ARR, annualized relapse rate; IVMP, intravenous methylprednisolone; IVIG, intravenous immunoglobulin; PE, plasma exchange; AZA, azathioprine; MMF, mycophenolate mofetil; TAC, tacrolimus; RTX, rituximab; CTX, cyclophosphamide. *p < 0.05, **p < 0.01, ***p < 0.001. [file Image_1.tif]
