# Supplementary material for: Neuromyelitis Optica Spectrum Disorder With Anti-Aquaporin-4 Antibody: Outcome Prediction Models
Source: Front Immunol. 2022 Mar 31;13:873576. doi: 10.3389/fimmu.2022.873576 (PMC9012141; doi:10.3389/fimmu.2022.873576)
Supplement: Supplementary file 2 [file Table_1.docx]

| **Table S1. Estimation of the effects of predictors on ARRs with univariate analysis** | | | | | | | | |
| --- | --- | --- | --- | --- | --- | --- | --- | --- |
| **Predictors** | **ON** | | **TM** | | **Brainstem/Cerebral** | | **All** | |
|  | **Rate ratio** | **p-value** | **Rate ratio** | **p-value** | **Rate ratio** | **p-value** | **Rate ratio** | **p-value** |
| Female gender (Reference = male) | 1.01 (0.94-1.09) | 0.75 | 1.07 (0.99-1.16) | 0.10 | 1.03 (0.98-1.08) | 0.25 | 1.10 (0.99-1.22) | 0.085 |
| AQP4-ab titer (Reference = < 1:100) | 1.04 (1.00-1.08) | 0.067 | 1.05 (1.00-1.09) | 0.033* | 0.99 (0.96-1.02) | 0.46 | 1.08 (1.02-1.14) | 0.011* |
| Onset age, years (Reference = > 48) |  |  |  |  |  |  |  |  |
| ≤ 35 | 1.03 (0.98-1.09) | 0.20 | 0.99 (0.94-1.04) | 0.68 | 1.04 (1.01-1.08) | 0.025* | 1.06 (0.99-1.14) | 0.099 |
| 35-48 | 1.01 (0.95-1.08) | 0.68 | 1.04 (0.98-1.11) | 0.20 | 1.00 (0.96-1.04) | 0.89 | 1.07 (0.98-1.16) | 0.11 |
| Onset attack (Reference = brainstem/cerebral) |  |  |  |  |  |  |  |  |
| ON | 1.13 (1.06-1.20) | <0.001*** | 0.95 (0.90-1.02) | 0.15 | 0.96 (0.92-0.99) | 0.025* | 1.02 (0.94-1.11) | 0.56 |
| TM | 1.00 (0.93-1.06) | 0.88 | 1.03 (0.97-1.11) | 0.32 | 0.91 (0.87-0.95) | <0.001*** | 0.95 (0.87-1.04) | 0.25 |
| Mixed | 1.03 (0.96-1.10) | 0.48 | 1.00 (0.92-1.07) | 0.91 | 0.97 (0.92-1.01) | 0.16 | 0.97 (0.88-1.07) | 0.51 |
| Concomitant auto-antibodies (Reference = < 1) | 0.96 (0.92-1.00) | 0.044* | 1.00 (0.95-1.04) | 0.87 | 1.00 (0.97-1.03) | 0.88 | 0.96 (0.90-1.01) | 0.13 |
| Concomitant auto-antibodies (Reference = < 2) | 0.97 (0.93-1.02) | 0.23 | 1.00 (0.95-1.05) | 0.94 | 0.99 (0.96-1.02) | 0.60 | 0.96 (0.90-1.03) | 0.25 |
| Onset EDSS score (Reference = < 2.5) | 1.02 (0.97-1.07) | 0.50 | 0.92 (0.88-0.97) | 0.003** | 0.97 (0.93-1.00) | 0.044* | 0.91 (0.85-0.97) | 0.005** |
| IVMP at the first attack (Reference = no) | 0.93 (0.89-0.98) | 0.009** | 0.96 (0.91-1.01) | 0.13 | 0.99 (0.96-1.03) | 0.63 | 0.91 (0.85-0.97) | 0.005** |
| Maintenance therapy (Reference = no or prednisone < 6 months) | 0.95 (0.89-1.01) | 0.11 | 0.97 (0.91-1.04) | 0.43 | 1.01 (0.97-1.06) | 0.50 | 0.94 (0.87-1.03) | 0.18 |
| AQP4-ab = anti-aquaporin-4 antibody; ON = optic neuritis; TM = transverse myelitis; EDSS = Expanded Disability Status Scale; EDSS = Expanded Disability Status Scale; IVMP = intravenous methylprednisolone. *p < 0.05, **p < 0.01, ***p < 0.001. | | | | | | | | |
