# Supplementary material for: Neuromyelitis Optica Spectrum Disorder With Anti-Aquaporin-4 Antibody: Outcome Prediction Models
Source: Front Immunol. 2022 Mar 31;13:873576. doi: 10.3389/fimmu.2022.873576 (PMC9012141; doi:10.3389/fimmu.2022.873576)
Supplement: Supplementary file 3 [file Table_2.docx]

| **Table S2. Estimation of the effects of predictors on certain events with univariate analysis** | | | | | | | | |
| --- | --- | --- | --- | --- | --- | --- | --- | --- |
| **Predictors** | **First relapse** | | **Blindness** | | **EDSS score ≥ 6.0** | | **EDSS score ≥ 8.0/Death** | |
|  | **Hazard ratio** | **p-value** | **Hazard ratio** | **p-value** | **Hazard ratio** | **p-value** | **Hazard ratio** | **p-value** |
| Female gender (Reference = male) | 1.28 (0.83-1.98) | 0.27 | 1.16 (0.54-2.52) | 0.70 | 0.99 (0.55-1.80) | 0.98 | 0.77 (0.35-1.69) | 0.52 |
| AQP4-ab titer (Reference = < 1:100) | 1.16 (0.92-1.46) | 0.21 | 1.13 (0.75-1.72) | 0.56 | 1.11 (0.80-1.55) | 0.53 | 0.93 (0.57-1.51) | 0.77 |
| Onset age, years (Reference = > 48) |  |  |  |  |  |  |  |  |
| ≤ 35 | 1.27 (0.94-1.72) | 0.12 | 0.41 (0.25-0.67) | <0.001*** | 0.69 (0.43-1.10) | 0.027* | 0.47 (0.27-0.83) | 0.009** |
| 35-48 | 1.24 (0.88-1.75) | 0.23 | 0.52 (0.29-0.91) | 0.022* | 0.64 (0.43-0.95) | 0.12 | 0.62 (0.33-1.19) | 0.15 |
| Onset attack (Reference = brainstem/cerebral) | |  |  |  |  |  |  |  |
| ON | 0.77 (0.56-1.07) | 0.13 | 4.66 (2.13-10.19) | <0.001*** | 0.53 (0.31-0.88) | 0.015* | 0.68 (0.28-1.65) | 0.39 |
| TM | 0.67 (0.47-0.96) | 0.028* | 0.72 (0.26-2.00) | 0.53 | 1.79 (1.11-2.89) | 0.016* | 2.45 (1.10-5.44) | 0.028* |
| Mixed | 0.69 (0.47-1.01) | 0.059 | 1.98 (0.80-4.92) | 0.14 | 1.20 (0.70-2.07) | 0.50 | 2.61 (1.14-5.97) | 0.023* |
| Concomitant auto-antibodies (Reference = < 1) | 0.95 (0.75-1.19) | 0.63 | 1.21 (0.80-1.84) | 0.36 | 1.42 (1.02-1.98) | 0.039* | 1.15 (0.71-1.87) | 0.57 |
| Concomitant auto-antibodies (Reference = < 2) | 0.79 (0.60-1.02) | 0.073 | 0.92 (0.58-1.47) | 0.73 | 1.36 (0.95-1.93) | 0.089 | 1.24 (0.74-2.08) | 0.42 |
| Onset EDSS score (Reference = < 2.5) | 0.70 (0.52-0.93) | 0.016* | 8.08 (5.29-12.34) | <0.001*** | 0.81 (0.53-1.23) | 0.32 | 1.56 (0.92-2.64) | 0.098 |
| IVMP at the first attack (Reference = no) | 0.59 (0.45-0.76) | <0.001*** | 0.62 (0.39-0.98) | 0.042* | 1.23 (0.82-1.85) | 0.32 | 1.72 (0.88-3.38) | 0.11 |
| Maintenance therapy (Reference = no or prednisone < 6 months) | 0.22 (0.14-0.34) | <0.001*** | 0.08 (0.04-0.18) | <0.001*** | 0.13 (0.07-0.24) | <0.001*** | 0.36 (0.17-0.76) | 0.007* |
| AQP4-ab = anti-aquaporin-4 antibody; ON = optic neuritis; TM = transverse myelitis; EDSS = Expanded Disability Status Scale; EDSS = Expanded Disability Status Scale; IVMP = intravenous methylprednisolone. *p < 0.05, **p < 0.01, ***p < 0.001. | | | | | | | | |
